# Migration of a Kirschner wire to the dorsolateral side of the foot following osteosynthesis of a patella fracture with tension band wiring: a case report

**DOI:** 10.1186/s13256-016-0819-5

**Published:** 2016-02-24

**Authors:** Ekrem Aydın, Turan Cihan Dülgeroğlu, Hasan Metineren

**Affiliations:** Department of Orthopaedics and Traumatology, Dumlupinar University School of Medicine, 43270 Kutahya, Turkey

**Keywords:** Kirschner wire migration, Patella fracture, Tension band

## Abstract

**Background:**

Patella fractures represent 1 % of all musculoskeletal system fractures. Fixation of patellar fractures using open reduction and tension band wiring is a commonly used and successful surgical fixation method.

**Case presentation:**

A 28-year-old male patient from Turkey presented to our clinic with complaints of palpable foreign bodies under the skin on the dorsolateral side of his right foot. Except for the palpable and moving body of about 6 cm length under the skin in his foot, he had no functional complaints. On X-ray, a Kirschner wire was visible in front of the lateral malleolus on the dorsolateral side of his right foot. In addition, there was a cerclage wire from the tension band fixation of his patella in the ipsilateral knee. The Kirschner wire was removed surgically.

**Conclusion:**

Despite the use of different fixation materials for the surgical treatment of patellar fractures, tension band wiring is still a commonly used technique. We recommend that after fixation of a patellar fracture using the tension band wiring technique, the ends of the Kirschner wires be bent and the wires then removed in the early phase after patellar union to prevent Kirschner wire migration.

## Background

Patella fractures represent 1 % of all musculoskeletal system fractures [1]. Most injuries caused by indirect trauma occur in the middle third part of the patella. Conservative treatment is carried out more frequently in patients with no incompatibility on the articular surface. Surgical treatment is performed in patients with 2–3 mm of articular displacement [2].

Arbeitsgemeinschaft für Osteosynthesefragen (AO)-modified tension band wiring is used in the treatment of fractures such as malleolus fractures, olecranon fractures, and transverse patella fractures [3–5]. Fixation of patellar fractures with open reduction and tension band wiring is a successful surgical fixation method commonly used today. Successful fixation can be achieved particularly in split patellar fractures. However, there may be Kirschner (K)-wire migration after fixation; to prevent this, the ends of the K-wire are generally bent and then the wires removed in the early phase after bone union [6]. Patellar fractures are frequently successfully treated using tension band wiring, and migration of the K-wire, as in our case, is limited. We believe this is the first report of pin migration to the dorsolateral side of the foot.

## Case presentation

**Fig. 1:**
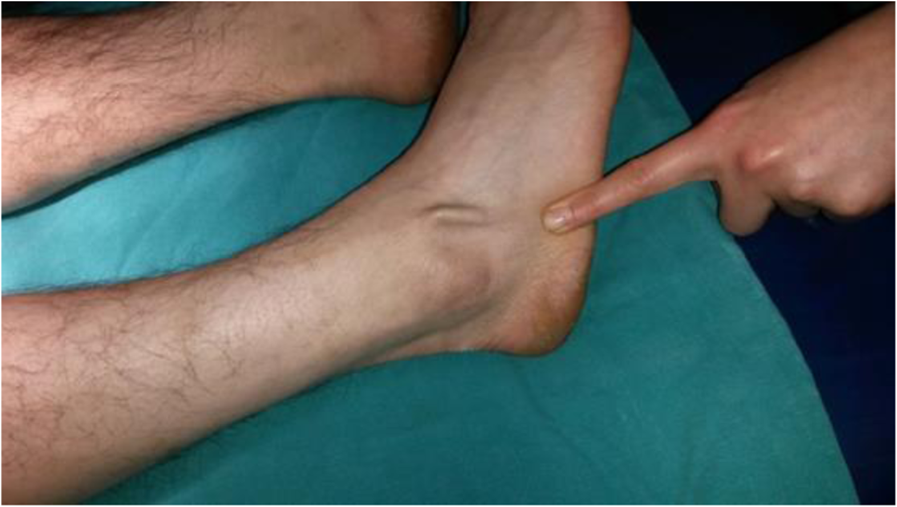
Palpable foreign body under the skin on the dorsolateral side of the right foot

**Fig. 2:**
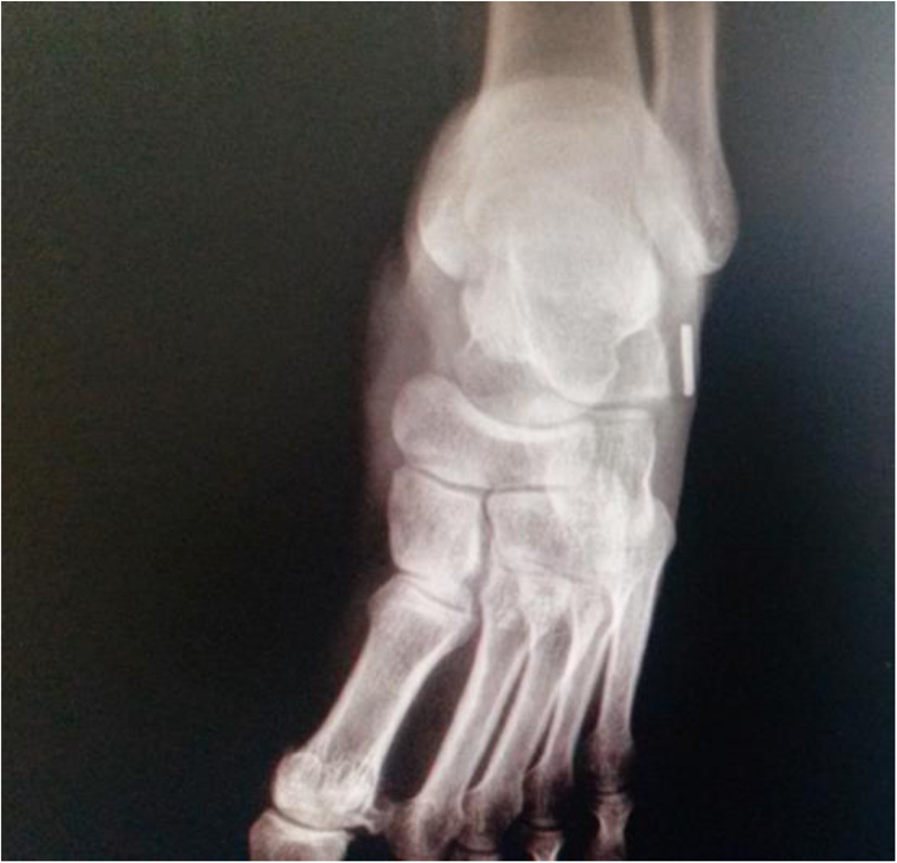
Anteroposterior plain radiography showing a Kirschner wire in the dorsolateral side of the right foot

**Fig. 3:**
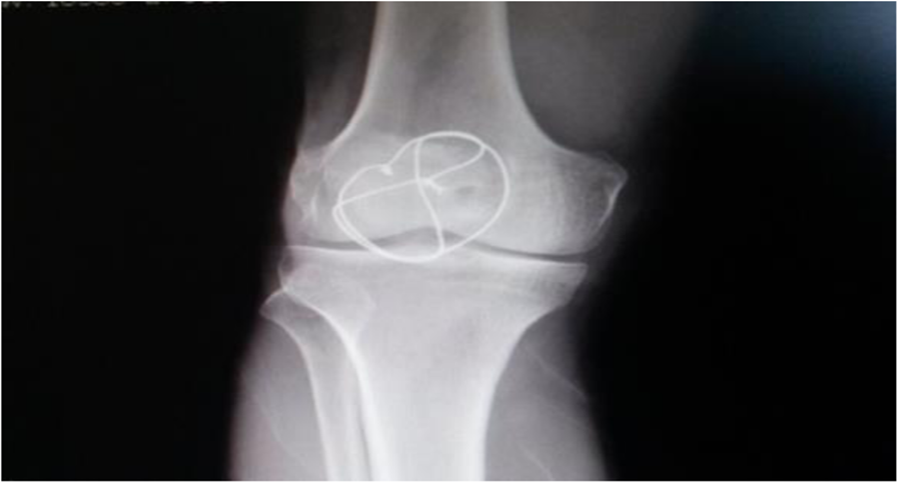
Anteroposterior plain radiography showing a cerclage wire from the tension band fixation of the patella of the ipsilateral knee

A 28-year-old male patient from Turkey presented to our clinic with complaints of palpable foreign bodies under the skin in the dorsolateral side of his right foot (Fig. 1). Except for the palpable and moving body of about 6 cm length under the skin in his foot, he had no functional complaints. An X-ray demonstrated the presence of a K-wire of about 6 cm length in front of the lateral malleolus in the dorsolateral side of his foot (Fig. 2). There was a cerclage wire from the tension band fixation of his patella in the ipsilateral knee (Fig. 3). There was no diagnostic challenge. Our patient explained that he was operated on by another orthopedic surgeon in the same hospital after a patellar fracture in 2007 and he had not attended regular checks. He also reported that a wire similar to the one in the X-ray had protruded through his skin in 2009 and he had removed the wire by pulling it out. This statement explained why the K-wire was not in place.

On physical examination, our patient showed no restriction in his knee movement. Routine blood test results showed no abnormalities. Palpation of the K-wire showed that the wire was mobile under his skin (Fig. 1). Our patient reported that, after removing the first wire, he had not felt any serious discomfort during the migration of the other wire over a period of 5 years.

**Fig. 4:**
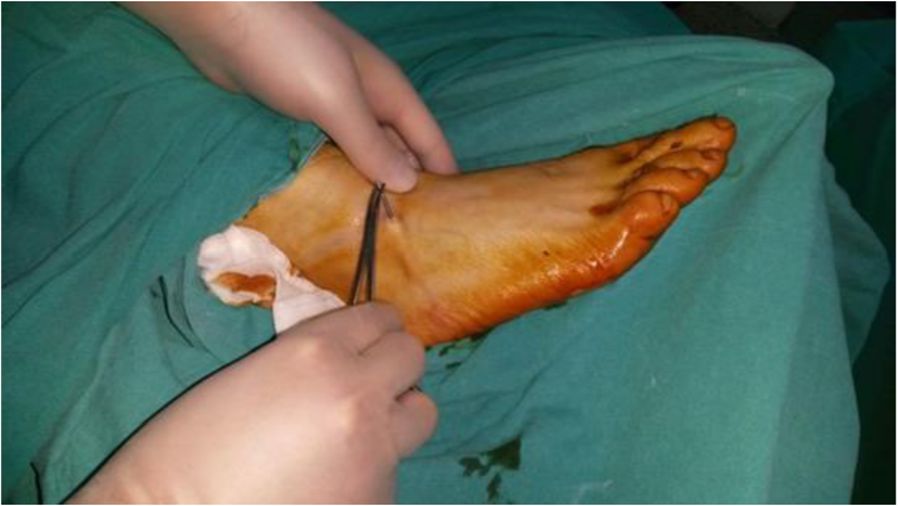
The Kirschner wire was pulled out with the help of a clamp

Our patient was taken to the operating room. After staining, a 3-mm skin incision was made that coincided with the proximal end of the K-wire, and the K-wire was pulled out with the help of clamp (Fig. 4). Our patient was discharged on the same day. The cerclage wire in his patella was not removed to avoid creating complications and because of our patient’s unwillingness to have it removed.

## Discussion

Despite the use of different fixation materials for the surgical treatment of patellar fractures, the tension band wiring technique is today still a commonly used method [7, 8]. Although a successful treatment option, if the ends of the K-wires are not bent after the operation and then removed in the early phase following bone union, wire breakages and various degrees of migration can be observed. Migrations in the popliteal and proximal anterior tibia after fixation of the patella using tension band wiring have been reported in the literature [7, 8]. Indeed, K-wire migrations have been reported after the fixation of fractures and dislocations in various regions. Longitudinal and lung migrations of the wire after the fixation of acromioclavicular and clavicle fractures are among the most frequently reported cases after migration following proximal humeral fracture fixation [9, 10]. Similarly, wire migration to the pelvis is among the complications reported after temporary transacetabular K-wire fixation to prevent dislocation during the cast winding process in developmental dysplasia of the hip [11]. It is possible for small, unnoticed wire fragments to remain in the tissue following wire removal and to be carried to distant regions, especially to the heart, after they penetrate the blood vessels [12]. Failure of hardware after pin migration is a well-known complication. Additionally, K-wires often break when retained for a long period after fracture union. Owing to their inherently smooth nature, K-wires are prone to migrate. Because they are thin, un-threaded, and tubular, K-wires have a strong tendency to migrate along the paths of least resistance. To prevent this, pins should be bent and left outside the skin in the majority of the patients [7]. We found few statistics in the literature about pin migration, although several literature reviews have been published, such as Lyons and Rockwood in 1990 (47 cases) [13], Freund *et al.* in 2007 (68 cases) [14], Sarma *et al.* in 2007 (four cases) [15], and Guèye *et al.* in 2015 (three cases) [16], suggesting that the number of cases of this complication is growing all over the world.

The migration of a K-wire from the patella to the dorsolateral side of the foot, as presented in this case, has not previously been reported in the literature. In our case, it is interesting that the K-wire was able to migrate to our patient’s foot without causing any symptoms or any additional complications.

## Conclusion

K-wires can easily migrate, which may result in significant complications. We recommend that the ends of the wires be bent, and the wires removed in the early phase after bone union to prevent K-wire migration following the fixation of a patellar fracture using the tension band wiring technique.

## Consent

Written informed consent was obtained from the patient for publication of this case report and accompanying images. A copy of the written consent is available for review by the Editor-in-Chief of this journal.

## References

[1] Lotke PA, Ecker ML. Transverse fractures of the patella. Clin Orthop Relat Res. 1981;158:180–4. 7273515

[2] Appel MH, Seigel H. Treatment of transverse fractures of the patella by arthroscopic percutaneous pinning. Arthroscopy. 1993;9:119–21. 10.1016/s0749-8063(05)80357-3 8442820

[3] Doursounian L, Prevot O, Touzard RC. Osteosynthesis by tension band wiring of displaced fractures of the olecranon. Ann Chir. 1994;48:169–77. 8192409

[4] Fan GF, Wu CC, Shin CH. Olecranon fractures treated with tension band wiring techniques – comparisons among three different configurations. Chang Gung Med J. 1993;16:231–8. 8313206

[5] Levack B, Flannagan JP, Hobbs S. Results of surgical treatment of patellar fractures. J Bone Joint Surg. 1985;67:416–9. 10.1302/0301-620X.67B3.3997951 3997951

[6] Kınık H, Us AK, Mergen E. Self-locking tension band technique: a new perspective in tension band wiring. Arch Orthop Trauma Surg. 1999;119:432–4. 10.1007/s004020050015 10613234

[7] Meena S, Nag HL, Kumar S, Borwar N. Delayed migration of K-wire into popliteal fossa used for tension band wiring of patellar fracture. Chin J Traumatol. 2013;16(3):186–8. 23735556

[8] Konda SR, Dayan A, Egol KA. Progressive migration of broken Kirschner wire into the proximal tibia following tension-band wiring technique of a patellar fracture--case report. Bull NYU Hosp Jt Dis. 2012;70(4):279–82. 23267457

[9] Karaman I, Kafadar IH, Oner M, Halici M. Intrapelvic pin migration after Salter innominate osteotomy and laparoscopic removal: a case report. J Pediatr Orthop B. 2013;22(6):553–5. 10.1097/BPB.0b013e32836422a0 23852036

[10] Yadav V, Marya KM. Unusual migration of a wire from shoulder to neck. Indian J Med Sci. 2003;57(3):111–2. 14514261

[11] Yurtçu M, Şenaran H, Türk HH, Abasıyanık A, Tuncay İ. Migration of intra-articular K-wire into the controlateral pelvis after surgery for developmental dysplasia of the hip: a case report. Acta Orthop Traumatol Turc. 2010;44(5):413–5. 10.3944/AOTT.2010.2334 21343694

[12] Sepicel RC, Schmeling GJ, Deley RA. Migration of a K-wire from the distal radius to the heart. Am J Orthop. 2001;30(2):147–51. 11234942

[13] Lyons FA, Rockwood Jr CA. Migration of pins used in operations on the shoulder. J Bone Joint Surg. 1990;72:1262–7. 2204634

[14] Freund E, Nachman R, Gips H, Hiss J. Migration of a Kirschner wire used in the fixation of a subcapital humeral fracture, causing cardiac tamponade: case report and review of literature. Am J Forensic Med Pathol. 2007;28:155–6. 10.1097/PAF.0b013e31806195a1 17525569

[15] Sharma H, Taylor GR, Clarke NM. A review of K-wire related complications in the emergency management of paediatric upper extremity trauma. Ann R Coll Surg Engl. 2007;89(3):252–8. 10.1308/003588407X155482 PMC1964701 17394709

[16] Guèye ML, Thiam O, Touré AO, Seck M, Cissé M, Kâ O, et al. Migration of guide pin in pelvic surgery in hip fractures: about 3 cases. Pan Afr Med J. 2015;21:112. 10.11604/pamj.2015.21.112.6541 PMC4546731 26327949

